# Identification of plasma exosomal lncRNA as a biomarker for early diagnosis of gastric cancer

**DOI:** 10.3389/fgene.2024.1425591

**Published:** 2024-10-08

**Authors:** Ye Wei, Xuming Hu, Shuai Yuan, Yue Zhao, Chunhui Zhu, Mingzhou Guo, Hengmi Cui

**Affiliations:** ^1^ College of Medicine, Yangzhou University, Yangzhou, China; ^2^ Institute of Epigenetics and Epigenomics and College of Animal Science and Technology, Yangzhou University, Yangzhou, China; ^3^ Yangzhou center for disease control and prevention, Yangzhou, China; ^4^ Department of Medical Affaires, Yangzhou Maternity and Child Health Hospital, Yangzhou, China; ^5^ Department of Gastroenterology and Hepatology, Chinese PLA General Hospital, Beijing, China

**Keywords:** gastric cancer, exosome, lncRNA, diagnosis biomarker, early diagonsis

## Abstract

**Background:**

There were about 1,090,000 gastric cancer (GC) cases in 2020 in China. The incidence and mortality rates ranked the fifth and third among all kinds of cancers in China. Early diagnosis plays an important role in the treatment and prognosis of gastric cancer. In recent years, noninvasive diagnosis, especially plasma exosome lncRNAs, has become a promissing biomarkers with high specificity and sensitivity for early diagnosis of cancers.

**Methods:**

In this study, plasma exosomes of patients with early gastric cancer were extracted efficiently by affinity membrane separation technology, including affinity adsorption, elution, affinity membrane regeneration and other steps. After identified by electron microscopy observation, particle size analysis and Western blot verification, the lncRNAs in the exosomes were extracted and were analysized by high-throughput RNA sequencing (RNA-Seq). The differentially expressed lncRNAs were verified by RT-qPCR in 93 patients with early gastric cancer and 49 normal controls.

**Results:**

Electron microscopy, particle size analysis and Western blot showed that exosomes were successfully isolated from plasma. RNA-Seq results show that 76 lncRNAs were upregulated and 260 lncRNAs were downregulated in plasma exosomes of early gastric cancer patients compared with normal controls. RT-qPCR analysis indicated that a total of 6 lncRNAs were significantly and differentially expressed in gastric cancer patients compared to normal controls, with 2 (lncmstrg. 1319590, Lncmstrg. 2312697) highly expressed and 4 lowly expressed (lncmstr-g.1004024.1, lncmstrg. 2441832.8, lncmstrg. 315376.1, lncmstrg.907985.2,) (*p* < 0.05). The survival curve analysis indicated that lncmstrg.2441832.8 and lncmstrg.2312697 had higher sensitivity and specificity for the diagnosis of gastric cancer, respectively and AUC curve areas were 0.6211 and 0.631, *p* < 0.05, respectively, which were greater than the traditional clinical detection indexes CEA (0.61) and AFP (0.57). When combined lncmstrg.2441832.8 and lncmstrg.2312697 in gastric cancer diagnosis, AUC curve area reached 0.73, which was greater than CA199 (0.71).

**Conclusion:**

Lncmstrg.2441832.8 and lncmstrg.2312697 may be a potential and promissing biomarkers for early diagnosis of gastric cancer.

## Introduction

Cancer is the second leading cause of human death in the world, and the number of deaths and morbidity are increasing year by year. Gastric cancer is one of the most important cancers that harm human health. According to 2020 cancer burden data released by IARC, the world’s newest gastric cancer cases are 1 million and 90 thousand cases and 770 thousand deaths in 2020. Incidence rate and mortality rate are the fifth and fourth of all kinds of tumors. China had 480 thousand new cases and 370 thousand deaths of gastric cancers and the incidence rate and mortality rate are the third of all kinds of tumors in the world (Latest global cancer data, 2020). At present, the specificity and sensitivity of commonly used tumor diagnosis markers such as CA199 and CEA are quietly low. Although gastroscopy is a gold standard for the diagnosis of gastric cancer, many people are unwell to do it because gastroscopy is an invasive examination. Additionally, the early symptoms of gastric cancer are hidden and difficult to detect. Therefore, these result in a low rate of early diagnosis of gastric cancer.

Exosome is an important subgroup of extracellular vesicles (Hill, 2017), which are usually considered as membranous vesicular bodies with a diameter of 30 ∼ 150 nm released to outside the cell through the fusion of multivesicles and cell membrane (Cui et al., 2018). It was found that exosomes exist in a variety of human fluids, such as serum (plasma), saliva, urine, amniotic fluid, milk, etc. (Vlassov et al., 2012).

Exosomes contain abundant bioactive molecules, such as proteins, mRNAs, lncRNAs and lipids, which participate in a variety of biological processes *in vivo*, such as intercellular material transport and signal transmission, angiogenesis, histone modification, immune activation/inhibition, cell growth and apoptosis. A large number of studies have shown that exosomes are closely related to the occurrence, growth, invasion, metastasis and metabolism of a variety of tumors.

lncRNAs are RNA molecules that do not encode or rarely encode proteins between 200 nt-100 kb in length (Yelin et al., 2003; Katayama et al., 2005; Bhan and Mandal, 2015). lncRNAs play a key role in the regulation of chromatin dynamics, gene expression, cell growth and differentiation (Qian et al., 2017). lncRNAs participate in a variety of cellular or biological processes by interacting with various biological macromolecules such as DNAs, proteins and RNAs (including mRNAs, microRNAs and other lncRNAs) (Functionality, 2017; Peng et al., 2018; Morlando and Fatica, 2018; Ang et al., 2018; Kopp and Mendell, 2018). lncRNAs have been identified to be involved in many complex cellular processes, such as cell death, growth, differentiation, apoptosis, epigenetic regulation, genomic imprinting, splicing, post transcriptional gene expression regulation, chromatin modification, inflammation, etc. (Hu et al., 2012; Flynn and Chang, 2014; Rossi and Antonangeli, 2014; Harries, 2012; Qian et al., 2017; Liu et al., 2016; Zhang et al., 2017; Yang et al., 2017). Recent studies have shown that some lncRNAs are highly expressed in various human cancers and play an important role in tumorigenesis, apoptosis, invasion and metastasis (Evans et al., 2016; Sitarz et al., 2018). In view of the high stability of exosome lncRNAs in body fluids, exosomal lncRNAs have a wide application potentiality in early cancer diagnosis.

## Materials and methods

### Patients

Ninety three plasma samples were collected from the patients with early gastric cancer in the Beijing 301 Hospital from 2018 to 2019 and 49 normal control plasma samples were obtained from the Jiangsu Oilfield General Hospital. All gastric cancer patients should be: 1) cancers by pathological diagnosis belong to early stage; 2) none received any cancer treatment. The following patients were excluded: 1) patients who were suffering from other cancers at the same time; 2) patients who were suffering from functional damage to their heart, liver and kidney. Clinical information was available for all gastric cancer patients and normal controls involved in the study. The study was approved by the Beijing 301 Hospital ethics committee, and all subjects signed the informed consent form. The plasma was isolated by centrifuged at 2,000 g for 10 min within 2 hours after obtaining the blood samples. The separated plasma was stored in a frezzer at - 80°C.

### Isolation of exosomes

1.5 mL plasma samples were first passed through a 0.8 µm diameter filter membrane (Millipore). Then, exosomes and exosomal RNA were isolateted according to the procedure of exoRNeasy MIDI Kit (Qiagen 77144). Briefly, add the sample into the exoEasy spin column and spin for 1 min at 500 g. Add 3.5 mL buffer XWP to the exoEasy Midi spin column, and spin 5 min at 5,000 g to wash the column and remove residual buffer, add 700 µL QIAzol to the membrane. Spin for 5 min at 5,000 g, briefly vortex the tube containing the lysate and incubate at room temperature for 5 min. The obtained exosomes were resuspended with 40 µL PBS and stored in −80°C freezer for further analysis.

### Identification of exosomes

#### NTA analysis

The concentration and particle size of exosomes were analyzed by Nanoparticle Tracking Analysis (NTA) with ZetaView (Particle Metrix, German).

### Transmission electron Microscopy (TEM) analysis

First the copper mesh containing 10 µL freshly isolated exosome samples were placed on the filter paper. After sucking the excess samples with the filter paper, the samples were dried in the air for 5 min. Then 1% uranyl acetate and negative dye were added on samples and standed for 2 min. After sucking the excess dye and drying in the air for 40 min, the samples were observed under transmission electron microscope (G2 F30 S-TWIN, FEI).

### Western blot

Exosomes were lysed in standard RIPA buffer supplemented with protease and phosphatase inhibitor cocktails (Roche). The amount of proteins was measured with a BCA protein assay kit (Beyotime, China). The protein was solubilized with loading buffer (5×) and heated at 100°C for 10 min. Proteins were separated by SDS-PAGE and then transferred to a 0.2-μm PVDF membrane (BioRad, United States). After blocking with Odyssey Blocking Buffer (Li-COR Biosciences, United States), the membrane was incubated with primary antibody (1:1,000, rabit anti-CD9, CD63) at 4°C overnight, then incubated with secondary antibodies (1:5,000, Goat anti-rabbit IgG).

### High throughput sequencing of exosomal RNAs

Plasma exosomal RNAs from 5 patients with early gastric cancer and 5 normal controls were used for high-throughput RNA sequencing. Exosomal RNAs were extracted using exorneasy MIDI Kit (Qiagen) and quantified with Nanodrop. The quality of RNA was assessed by capillary electrophoresis on an Agilent 2,100 Bioanalyzer (Agilent Technologies, CA). High throughput RNA sequencing was completed by Baimike company (Beijing) and RNA sequencing was done using Illumina hiseq platform.

### Real-time quantitative PCR

Purified RNA was reversely transcribed into cDNA using the Hiscript Q RT SuperMix. Then, qRT-PCR was performed using SYBR Green assays (Vazyme) on a BIO-RAD CFX Connect system. The reactions were incubated at 95°C for 10 min, followed by 45 cycles of 95°C for 5 s and 60°C for 30 s. All experiments were conducted in triplicate, and the products were confirmed by melting curve analysis following each reaction. GAPDH RNA levels were used as internal control to normalize gene expression. The CDS sequences of differentially expressed lncRNA were obtained by gene sequencing, and the 5′-RACE and 3′RACE specific primers were designed using Primer 6.0 and synthesized by GENEWIZ Company. The primers used for PCR are as followings (Table 1):

**TABLE 1:** Primers used in this study.

Gene product	Primer sequence
lncMSTRG.1319590	Forward primer: AGA​GTC​TCG​TTC​GTT​ATC​G
	Reverse primer: CGG​ACA​GGA​TTG​ACA​GAT​T
lncMSTRG.2312697	Forward primer: TCC​ATC​CAT​CCA​TCA​TCT​ATC
	Reverse primer: ATG​CTG​GAT​GAA​TGG​AGA​AT
lncENSG00000095932	Forward primer: TCC​ATC​CAT​CCA​TCA​TCC​A
	Reverse primer: AGTGGGTGAATGGGTGAG
lncENSG00000182162	Forward primer: ATG​AAT​GAG​TGA​GTG​AAT​GG
	Reverse primer: CAT​CCA​TCC​ATC​CAT​CCA​T
lncENSG0000014881	Forward primer: CAC​TTA​CAC​CCA​CCC​TTA​C
	Reverse primer: GGCGTGGAGGTAGATGTA
lncENSG00000214226	Forward primer: CACCATCAGCACCATCAC
	Reverse primer: TTG​TGG​TGG​TGG​TAA​TAG​TG
GAPDH	Forward primer: CCG​GGA​AAC​TGT​GGC​GTG​ATG​G
	Reverse primer: AGG​TGG​AGG​AGT​GGG​TGT​CGC​TGT​T

The relative expression levels of the lncRNAs were calculated using the 2^−ΔΔCT^ method.

### Statistical analysis

All statistical analyses were performed with SPSS 22.0 and GraphPad Prism 8.0 Software. The difference of the expression levels of plasma exosomal lncRNAs between GC patients and normal controls were evaluated by a Student’s *t*-test or one-way ANOVA. Receiver operating characteristic curve (ROC) and area under curve (AUC) were used to estimate the diagnostic value of each index for GC. A combined ROC was calculated on the basis of the logistic regression model. *P* < 0.05 was regarded as statistically significant.

## Results

### Characteristics of plasma exosomes

**FIGURE 1:**
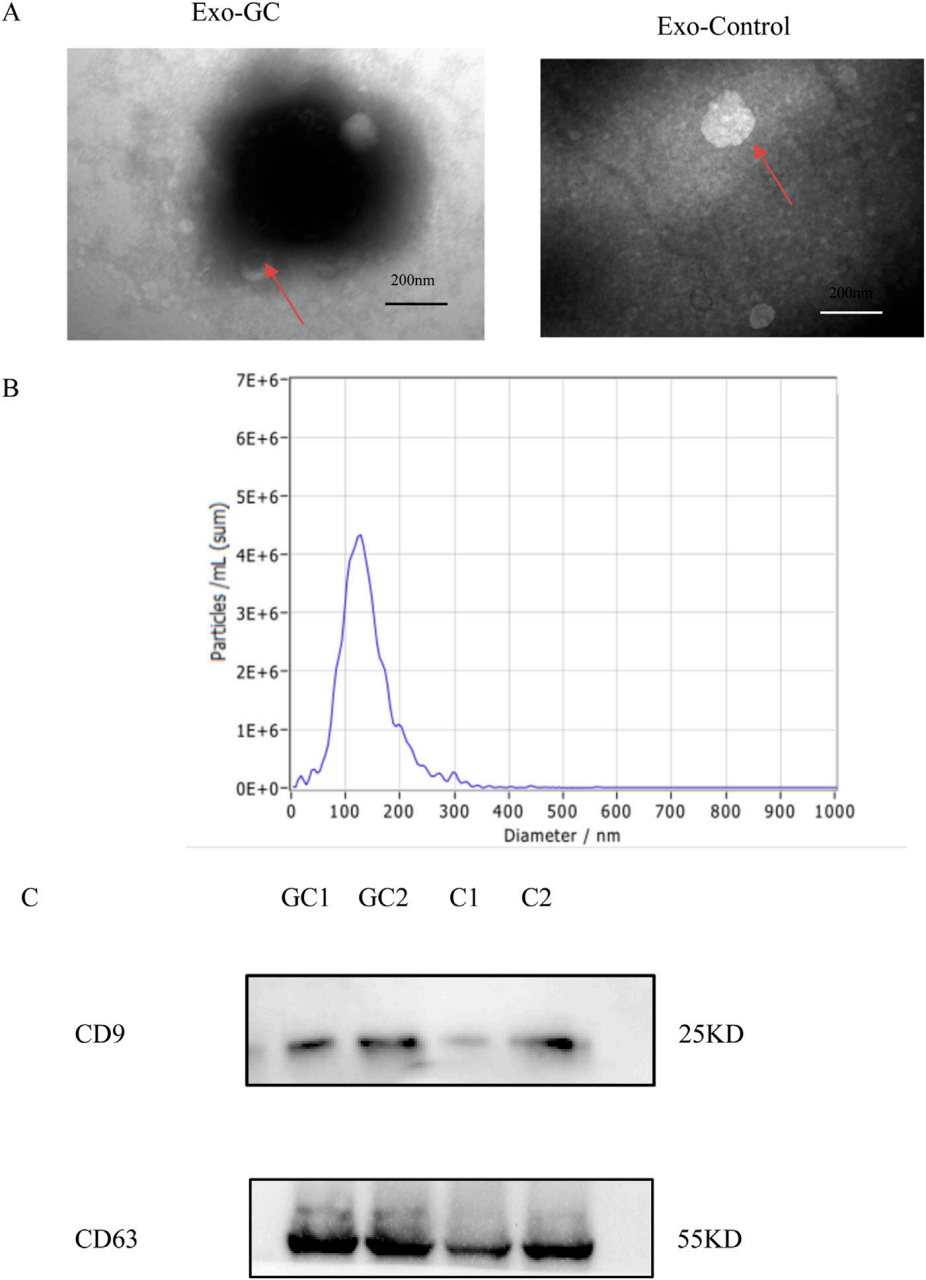
Identification of purified exosomes. **(A)** Representative transmission electron microscopy image of purified exosomes from culture medium (Exo-GC) and plasma (Exo-Control) (scale bar, 200 nm). **(B)** NTA of the size distribution and number of exosomes. **(C)** Western blot analysis of CD9 and CD63 in plasma exosomes.

We successfully extracted relatively pure plasma exosomes through the kit, and identified the isolated exosomes by transmission electron microscope, particle size analysis and Western blot. Exosomes with typical “cup cap” morphology can be directly observed by projection electron microscope (see Figure 1A). The particle size analysis shows that the average diameter of exosomes is 128.8 nm, which is in line with the diameter range of exosomes obtained in various current studies (Figure 1B). Western blot showed that both marker proteins CD9 and CD63 were expressed in exosomes (see Figure 1C), which was consistent with the characteristics of exosomes. The above results show that we have successfully isolated exosomes from plasma.

### Screening of differentially expressed exosomal lncRNAs in early gastric cancers by high throughput RNA sequencing assay

**FIGURE 2:**
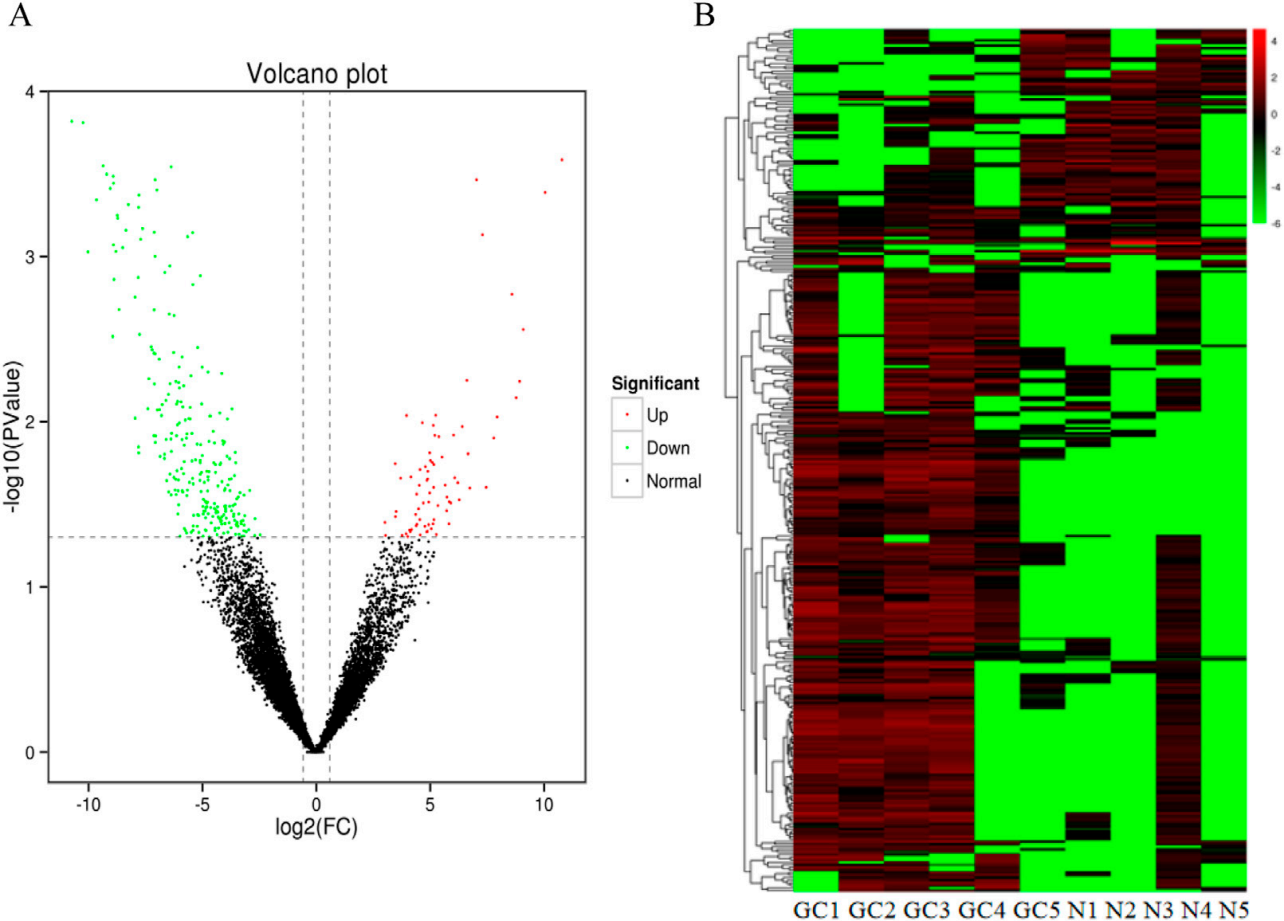
Identification of differentially expressed lncRNAs by RNA-seq. **(A)** A total of 336 differential expression lncRNAs were obtained between GC groups and normal controls **(B)** Heatmap result of differential expression lncRNAs between GC groups and normal control based on RNA-seq. Different columns in the figure represent different samples, and different rows represent different lncRNAs. The color represents the lncRNA expression levels.

We extracted plasma exosomal RNA from 5 patients with early gastric cancers and 5 normal controls for high-throughput RNA sequencing assay. The sequencing results showed that 76 lncRNAs were upregulated and 260 lncRNAs were downregulated compared with the normal controls (fold change≥ 1.5, *P* < 0.05). The difference of lncRNA expression levels between gastric cancers and controls can be seen by volcano map and heat map.It can be found that lncRNA expression in early gastric cancer group is higher than that in controls group (Figure 2). Seven lncRNAs with the largest expression difference were selected for the further verification.

### Verification of selected differentially expressed exosomal lncRNAs in plasma samples of gastric cancer patients

**FIGURE 3:**
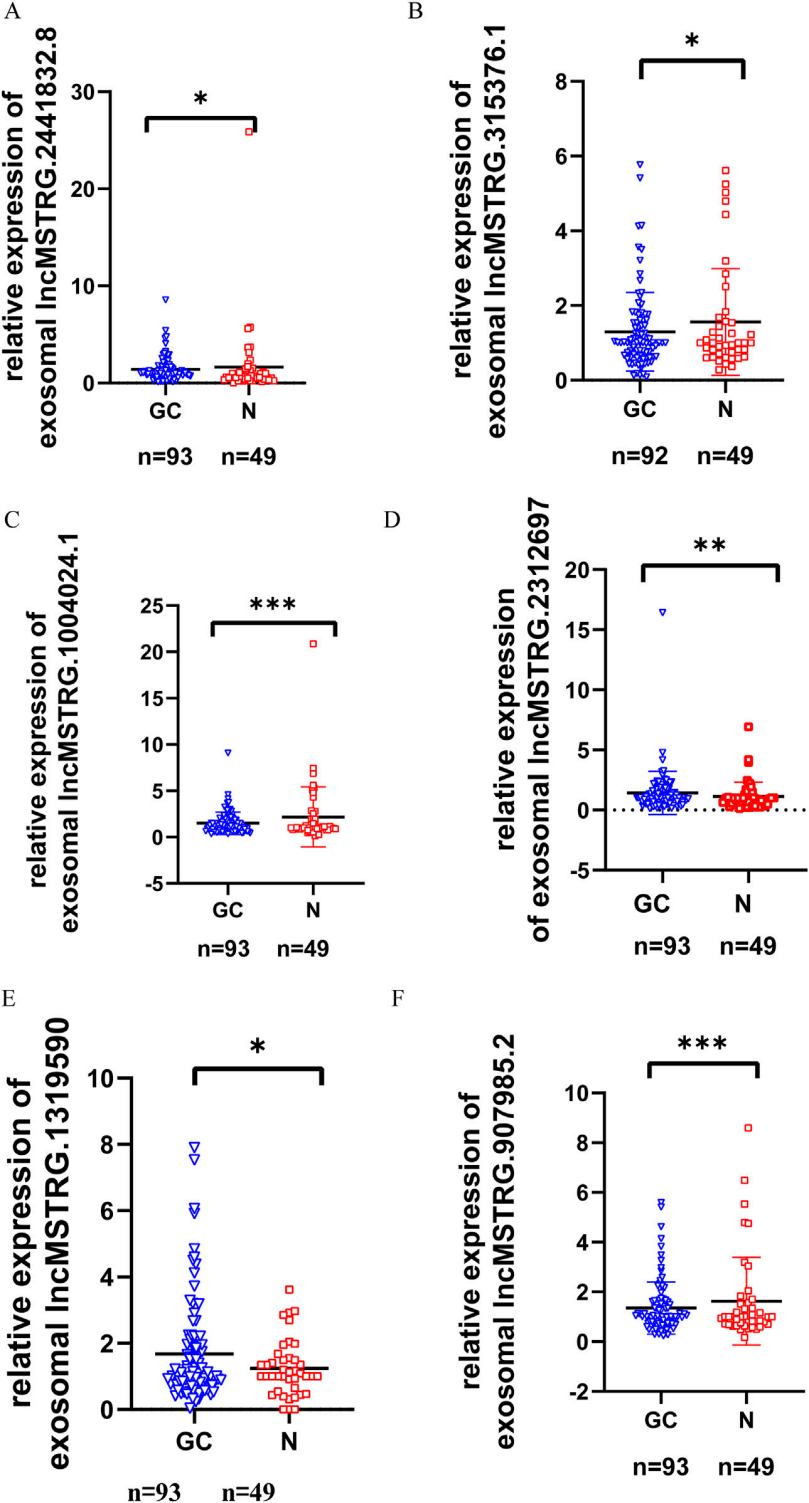
Relative expression levels of 6 exosomal lncRNAs in GC patients and normal controls were validated in clinical samples. Relative expression levels of 79 exosomal lncRNAs were examinated by RT-qPCR in GC patients and normal controls. Vertical axis represents relative gene expression 2^-△△Ct. **(A)** Expression levels of lncMSTRG.2441832.8 in GC patients and normal control. **(B)** Expression levels of lncMSTRG.315376.1 in GC patients and normal control. **(C)** Expression levels of lncMSTRG.1004024.1 in GC patients and normal control. **(D)** Expression levels of lncMSTRG.2312697 in GC patients and normal control. **(E)** Expression levels of lncMSTRG.1319590 in GC patients and normal control. **(F)** Expression levels of lncMSTRG.907985.2 in GC patients and normal control.

To verify the differential expression of exosomal lncRNAs in early gastric cancer patients, plasma exosomes and RNA were isolated from 93 patients with early gastric cancer and 49 normal controls (In this study, differentially expressed genes were defined as those with a change in gene expression level of >1.5 and *p* ≤ 0.01 between the normal control and the gastric cancer group). Seven lncRNAs with the largest differential expression (The differentially expressed multiples ranged from 5 to 13) were selected from 336 differential expression lncRNAs. These lncRNAs were further confirmed by RT-qPCR (Figures 3A–F). The results indicated that 6 lncRNAs showed significant differences in the expression levels between the gastric cancers and the controls (*P* < 0.05), of which 2 lncRNAs (lncmstrg.1319590 and lncmstrg.2312697) were highly expressed and 4 were lowly expressed in the plasma exosomes of gastric cancer patients (lncmstrg.1004024.1, lncmstrg.2441832.8, lncmstrg.315376.1, lncmstrg.907985.2).

### The verification of differentially expressed exosomal lncRNAs in clinical samples

**FIGURE 4:**
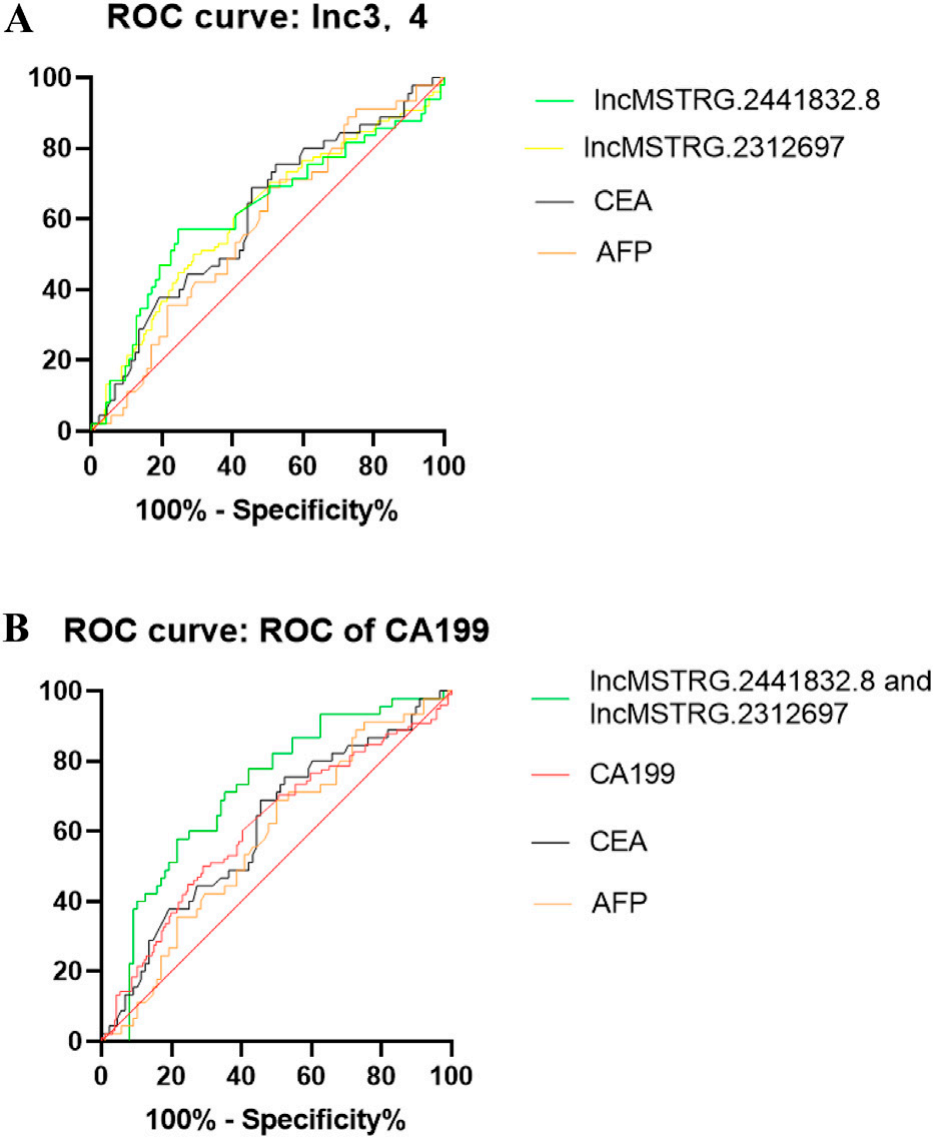
ROCs of plasma exosomal lncMSTRG.2441832.8, lncMSTRG.2312697 compared with traditional biomarkers CEA, AFP and CA19-9. **(A)** ROCs of plasma exosomal lncMSTRG.2441832.8, lncMSTRG.2312697 and plasma CEA, AFP in GC patients versus healthy controls. **(B)** ROCs of the combination of plasma exosomal lncMSTRG.2441832.8 with lncMSTRG.2312697, compared with plasma CEA, CA19-9, and AFP in GC patients versus healthy controls.

Based on the six differentially expressed lncRNAs in the plasma exosomes of early gastric cancer patients with the largest difference, we used the receiver operating characteristic curve to calculate the specificity and sensitivity of the differentially expressed lncRNAs for the early diagnosis of gastric cancer and decided whether they would have diagnostic advantage compared with the traditional tumor markers like CEA, CA199 and AFP. ROC results indicated that lncmstrg.2441832.8 and lncmstrg.2312697 had higher sensitivity and specificity for the diagnosis of gastric cancer, respectively. The Area Under Curves were 0.6211 and 0.631 (*P* < 0.05), while AUCs of CEA and AFP were 0.61 and 0.57, respectively. When we combined lncmstrg.2441832.8 with lncmstrg.2312697 to diagnose early gastric cancer, the AUC reached 0.73, was greater than 0.71 of CA199 (Figures 4A, B). These results indicate that lncmstrg.2441832.8 and lncmstrg.2312697 may be used as potential biomarkers for early diagnosis of gastric cancer.

## Discussion

Gastric cancer is one of the common malignant tumors endangering human health. Because the current commonly used tumor markers cannot accurately diagnose gastric cancer, especially early gastric cancer, gastric cancer is usually found in the middle and late stage, which seriously affects the treatment and prognosis of patients. The 5-year survival rate is usually only 10%–30% (Jeddi et al., 2018)

The development of liquid biopsy technology provides a technical means for early detection of tumor specific markers in patients’ blood. Its noninvasivity, sensitivity and accuracy bring new hope for early tumor diagnosis. After early detection and standardized treatment, the 5-year survival rate of gastric cancer can reach more than 90%. Exosome detection is an important part of liquid biopsy technology. Exosomes are vesicle like bodies actively secreted by a variety of living cells. There are a variety of bioactive substances in exosomes, including DNAs, proteins, mRNAs, miRNAs, lnRNAs, etc. Exosomes are representative of source cells and can carry specific biological macromolecules of source cells, including tumor cells. It can reflect the pathophysiological state of tumor patients and the nature of primary tumors, and is expected to be used in the clinical diagnosis of malignant tumors (Clayton and Mason, 2009).

Exosome encapsulated lncRNAs have good stability in blood and is usually considered as a biomarker for early diagnosis of gastric cancer. Studies have shown that the high expression of hottip in serum exosomes is positively correlated with tumor size, pathological stage, metastasis and prognosis of patients with gastric cancer. Hottip can mediate cell proliferation by inhibiting p21 or leading to miRNA silencing. The expression of hottip in plasma exosomes of patients with gastric cancer was significantly increased and the AUC was 0.827, which was significantly higher than clinical biomarkers such as CEA and CA199 (Zhao et al., 2018).Through sequencing analysis of plasma exosomal RNA, it was found that plasma exosomal lnceegc1 was significantly upregulated in patients with early gastric cancer, and the AUC was 0.84, which was much higher than conventional markers such as CEA (Lin et al., 2018). In addition, linc00152, zfas1, ufc1 and hotair in plasma (serum) exosomes have also been proved to be potential biomarkers for early diagnosis of gastric cancer.

In this study, 336 differentially expressed lncRNAs were obtained by high-throughput sequencing analysis of RNAs in exosomes of early gastric cancers and normal controls, of which 76 were upregulated and 269 were downregulated in gastric cancer samples. The Gene Ontology (GO) enrichment analysis revealed that the differentially expressed genes in gastric cancer were primarily associated with molecular functions such as protein binding, cell surface protein localization, DNA metabolism, and inhibition of protein degradation. These genes were predominantly localized in the cytoplasm and exosomes. Furthermore, the biological processes involve encompassed apoptosis, transcriptional regulation, GTP energy activity, heme binding, etc. Additionally, KEGG analysis demonstrated that the differentially expressed genes were mainly implicated in metabolic pathways like cell endocytosis and platelet activation as well as signaling pathways including phospholipase D signaling pathway and focal adhesion signaling pathway.

Seven lncRNAs with the largest differential expression changes were selected for further verification, and further 6 differentially expressed lncRNAs, including 2 with higher expression and 4 with lower expression were obtained. Through ROC analysis, we infered that lncmstrg.2441832.8 and lncmstrg.2312697 can be used as lncRNA biomarkers for early diagnosis biomarkers of gastric cancer. To our knowledge, the above two potential lncRNA biomarkers have not been reported so far. Their biological functions and target genes remain to be further studied. Additionally, the number of gastric cancer and controls included in this study is not enough. We need to increase the sample size to deeply analyze the diagnostic specificity and sensitivity of lncmstrg.2441832.8 and lncmstrg.2312697.

In conclusion, RNA genome sequencing of plasma exsomes from gastric cancer patients was performed to screen RNA candidate markers for gastric cancer. We further identified two exosomal lncRNA biomarkers that may are expected to be used as potential biomarkers for early diagnosis of gastric cancer. In the future, a validation experiment remain to be performed in a larger number sample from gastric cancer patients to obtain a molecular assemblage of RNA markers for early diagnosis of gastric cancer.

## Data Availability

The data presented in the study are deposited in the NCBI repository, accession number PRJNA1166350.
